# Evaluation of thymidine kinase 1 and folate receptor alpha as potential biomarkers in prostate cancer

**DOI:** 10.1038/s41598-025-15375-0

**Published:** 2025-08-19

**Authors:** Sarah Ibrahim Ahmed, Abeer Mohamed Mohy, Hossam Mohamed Farid, Ghada Nabil Elnaggar

**Affiliations:** 1 Department of Clinical Pathology, National Cancer Institute, Cairo University, Cairo, Egypt; 2 Department of Clinical and Chemical Pathology, Cairo University, Cairo, Egypt; 3 Surgical Oncology Department, National Cancer Institute, Cairo University, Cairo, Egypt

**Keywords:** Prostate cancer, TK1, FORα, Biochemistry, Cancer

## Abstract

Prostate cancer is a widespread malignancy with high mortality rates. Therapeutic success depends on clinical biomarkers for early detection and progression of the disease. The study aimed to assess the potential role of Thymidine Kinase one (TK1) and folate receptor alpha (FORα) in prostate cancer. Forty-five newly diagnosed prostate cancer patients, and 45 age matched apparently healthy males were included. Serum levels of TK1 and FORα were measured using enzyme-linked immunosorbent assay (ELISA) technique. TK1 and FORα were significantly increased in prostate cancer patients compared to the control group (mean 28.11pg/ml vs. 15.66 pg/ml, p-value < 0.001) and (median 1686.4 pg/ml vs. 437.2 pg/ml, p-value < 0.001) respectively. At a cutoff level ˃22.1 pg/ml, TK1 showed 91.11% sensitivity, 88.89% specificity and AUC = 0.973 (95% CI: 0.945–0.999). FORα showed moderate discriminatory power with 73.33% sensitivity and 88.89% specificity, AUC was 0.770 (95% CI: 0.661–0.879) at cutoff level ˃918.16 pg/ml. Combining TK1 with total PSA markedly improved the diagnostic accuracy (sensitivity 95.56%, specificity 97.78%). TK1 showed significant positive correlation with Gleason score, WHO grade and presence of metastasis at *p* < 0.001 each. TK1 & FORα could serve as biomarkers for prostate cancer diagnosis and prognosis offering a novel treatment approach.

## Introduction

Prostate cancer is well known for having a high incidence rate and a high degree of heterogeneity. According to reports, the disease caused 397,430 deaths globally and roughly 1,467,854 onsets in 2022, leaving a massive burden on society. It is the fourth most prevalent cancer, after lung, breast, and colorectal cancers^1^.

Prostate cancer follows a prolonged and complex disease course, which is classified based on factors such as prostate-specific antigen (PSA) levels, Gleason score (GS), TNM staging, and clinical presentation. These classifications help define disease stages, including localized, advanced, and metastatic prostate cancer, each requiring distinct therapeutic strategies^2^.

For both detection and monitoring, PSA is the gold standard serum biomarker for prostate cancer. However, some patients with poorly differentiated tumours have low PSA levels despite having advanced disease. Therefore, PSA value is limited in more advanced stages of prostate cancer^3,4^.

One of the hallmarks of malignant tumours is unchecked cell proliferation. The clinical progression of various neoplasms is largely dependent on the proliferative activity of neoplastic cells. Therefore, indicators of proliferation become attractive candidates as tumour biomarkers^5^.

Thymidine kinase 1 and folate receptor alpha are two of these recognized markers. Thymidine kinase 1 (TK1) is an enzyme which is involved in the synthesis of deoxyribonucleic acid (DNA). As a step in the DNA synthesis process, thymidine is phosphorylated by thymidine kinases. Intracellular concentrations of TK1 are low throughout the G0/G1 phases of the cell cycle and increase during the S/G2 phases, indicating active cellular proliferation. Accordingly, its serum concentration reflects the rate of proliferation and the cell death extent. Serum TK1 levels have been proposed as a potential marker for the presence of prostate cancer in a few prior investigations and has been investigated as a monitoring and prognostic marker in leukemia, Hodgkin and non-Hodgkin lymphomas^4,6^.

Folate receptor alpha (FORα) is a glycosylphosphatidylinositol (GPI)-anchored cell-surface glycoprotein that has a strong affinity for folic acid and its derivatives. It works with signalling cascades and folate cycle components to mediate various cellular processes, such as cell division, proliferation, and tissue growth. Although FORα is not widely distributed in non-malignant tissues, it has been found to be highly expressed in a number of malignancies, such as ovarian, endometrial, lung, and breast tumours. Tumour cells have the ability to release FORα, which can be found in the bloodstream in a soluble form^7^.

The aim of this study was to assess the serum TK1 and FORα expression in prostate cancer patients by ELISA and to correlate their levels with the severity of the disease, as well as studying their correlation with another well-documented prostate cancer marker (PSA) for a possible higher diagnostic accuracy through improved sensitivity and/ or specificity.

## Materials and methods

### Patients

This cross-sectional study was carried out through the course of 7 consecutive months from January 2024 to July 2024. Serum samples were obtained from 45 patients with different stages of prostate cancer, all presented to the surgical oncology outpatient clinics, National Cancer Institute (NCI), Cairo University as well as 45 age matched apparently healthy individuals as controls. Subjects were classified into two groups:

Group (1): Prostate cancer group including 45 adult male patients diagnosed as prostate cancer with no other malignancies with range of age between 46 and 82 years (mean age 67.91 years ± 8.82 SD).

Group (2): Control group including 45 age matched healthy males with no given history of malignant diseases with range of age 50 to 80 years (mean age 66.96 years ± 7.83 SD).

Written informed consents were obtained from all participants prior to enrolment in the study. The study was approved by the ethical committee, and the review board of NCI, Cairo University in accordance with Helsinki guidelines for the protection of human subjects (Approval No. CP2307-403–031). Patients’ characteristics are presented in Table 1.

**Table 1:** Individual characteristics of prostate cancer patients.

Mean age	67.91 years ± 8.82	
	*N*	%
Pathology		
Adenocarcinoma	44	97.8%
Leiomyosarcoma	1	2.2%
Gleason score		
Score 6	4	9.1%
Score 7	12	27.3%
Score 8	16	36.4%
Score 9	12	27.3%
WHO grade		
Grade I	4	9.1%
Grade II	5	11.4%
Grade III	7	15.9%
Grade IV	16	36.4%
Grade V	12	27.3%
Metastasis		
Yes	28	62.2%
No	17	37.8%
Family history		
Yes	2	4.4%
No	43	95.6%

Data are represented as the number of cases (N) and percentage (%).

### Methodology

All patients in this study were subjected to full history taking focusing on age, family history, clinical background and comorbidities. Imaging studies were done in the form of trans-rectal ultrasound (TRUS), PET scan, CT and MRI scan of pelvis, abdomen and chest to record any metastatic findings related to the disease. Prostate cancer was pathologically confirmed and graded using Gleason’s score and WHO grade group. Regarding laboratory investigations; all patients and controls performed Total and Free PSA (measured by electro-chemiluminescence on Cobas e801 auto-analyser), and quantitative measurement of serum levels of TK1 and FORα by Enzyme Linked Immunosorbent Assay (ELISA) kits.

### Specimen collection and Preparation

Peripheral venous samples 5 ml were obtained from all subjects prior to any surgical or medical intervention, under complete aseptic precautions and left for 15 min at room temperature to clot, then centrifuged at a speed of 3000×*g* for 5 min. The serum was then separated into Eppendorf tubes and stored at − 20 °C until time of assay.

Thymidine Kinase 1 was done using Human soluble TK1 ELISA kit (ELKBiotech Co., Ltd. Catalog no.: ELK3552) according to manufacturer’s instructions. Folate Receptor alpha was done using Human FORα kit (ELKBiotech Co., Ltd. Catalog no.: ELK3046) according to manufacturer’s instructions.

### Statistical analysis

The collected data was revised, coded, and tabulated using Statistical package for Social Science (IBM Corp. Released 2017. IBM SPSS Statistics for Windows, Version 25.0. Armonk, NY: IBM Corp.).

Data were statistically described in terms of mean ± standard deviation (± SD), median and range, or frequencies (number of cases) and percentages when appropriate. Shapiro-Wilk test was done to test the normality of data distribution. Comparison of numerical variables between the study groups was done using Mann Whitney *U* test for independent samples. Student T Test was used to assess the statistical significance of the difference of parametric variable between two study group means. Correlation analysis was done to assess the strength of association between two quantitative variables.

Accuracy was represented using the terms sensitivity, and specificity. Receiver operator characteristic (ROC) analysis was used to determine the optimum cut off value for the studied diagnostic markers.

Logistic regression analysis was used for the prediction of risk factors when the dependent variable is categorical. Odds ratio (OR) and 95% confidence interval (CI) were calculated. Two-sided *p* values less than 0.05 was considered statistically significant.

## Results

### Comparison of serum total PSA, TK1 and FORα for prostate cancer diagnosis

**Fig. 1:**
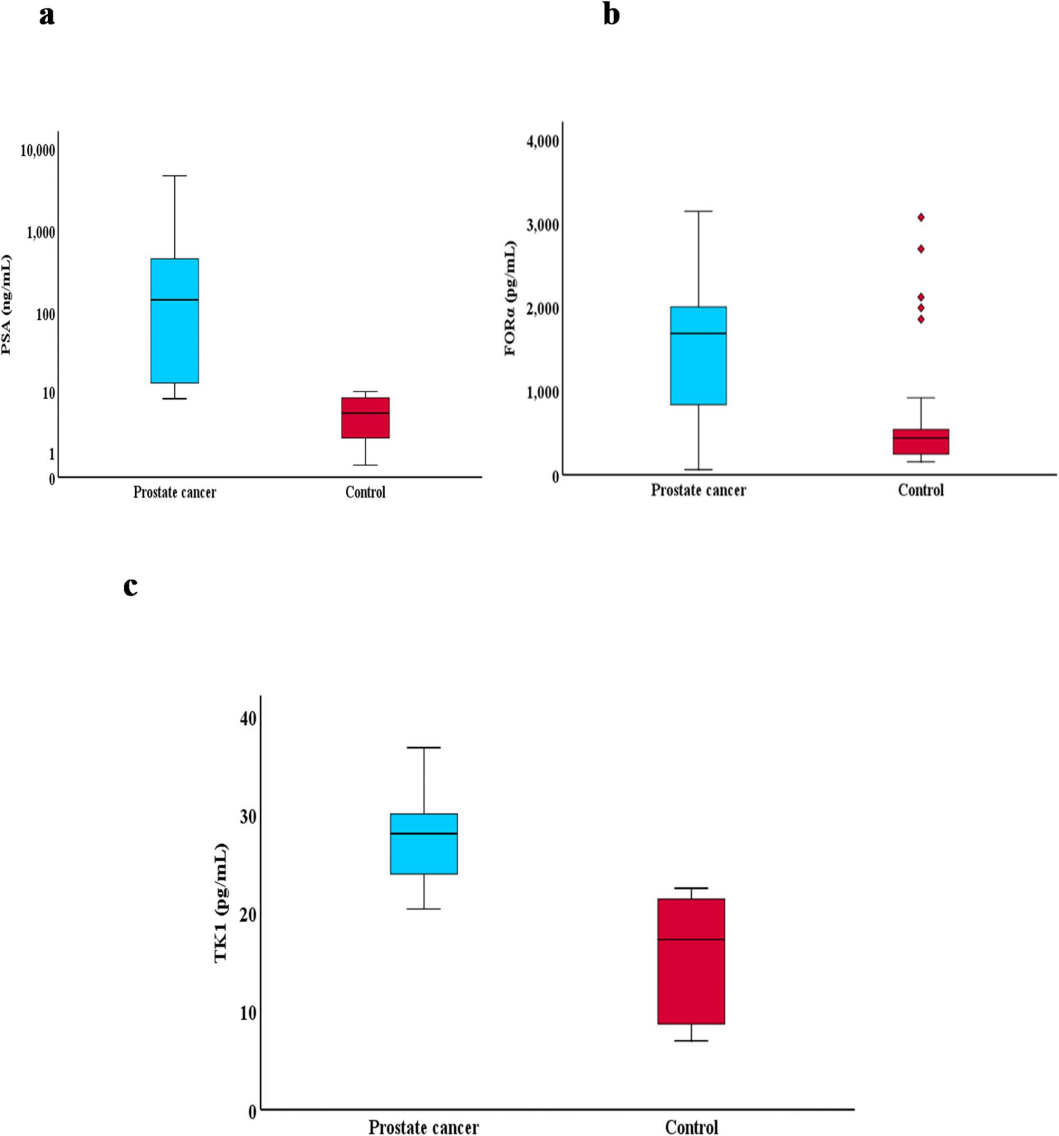
Boxplot chart for comparison between patients with prostate cancer and the control group regarding total PSA (**a**), FORα (**b**) and TK1 (**c**).

All three markers were significantly higher in prostate cancer patients compared to the control group with P value < 0.001 each as shown in Table 2; Figure 1.

**Table 2:** Comparison between prostate cancer group and the control group regarding serum total PSA, TK1 and FORα.

	Group 1 *n* = 45	Group 2 *n* = 45	*P*
Total PSA (ng/mL)			
Median	142.69	5.0	< 0.001*
Min.–Max.	8.0–4640.0	0.40–10.0	
TK1 (pg/mL)			
Mean ± SD	28.11 ± 4.41	15.66 ± 5.89	< 0.001*
Min.–Max.	20.40–36.80	7.0–22.50	
FORα (pg/mL)			
Median	1686.4	437.2	< 0.001*
Min.–Max.	62.0–3142.0	156.0–3070.0	

*SD.* standard deviation, *Min.* minimum, *Max.* maximum, Student t test& Mann Whitney test.

p: Comparing the two studied groups, *: Significant when p value < 0.05.

### Association between total PSA, TK1, FORα and different parameters among patients with prostate cancer

Our results showed significant differences between Gleason scores, WHO grades and metastasis regarding total PSA and TK1 levels among patients with prostate cancer. Overall, there was trend towards elevation of total PSA as well as TK1 with higher Gleason scores, higher grades of tumour aggressiveness and presence of metastasis, with p-values shown in Table 3.

**Table 3:** Association between serum total PSA, TK1 and FORα with Gleason score, WHO grade and metastasis among prostate cancer patients.

Variable	No.	%	Total PSA ng/ml Median (Min-Max)	*P*	TK1 pg/ml Mean ± SD	*P*	FORα pg/ml Median (Min-Max)	*P*
Gleason Score								
Score 6	4	9.1	13.20 (9.00–98.47)	0.009*	22.84 ± 1.39	< 0.001*	930.37 (149.19–2002.57)	0.415
Score 7	12	27.3	13.00 (8.00–4640.00)		25.27 ± 3.05		1910.82 (62.00–2705.29)	
Score 8	16	36.4	299.63 (8.60–975.00)		27.85 ± 2.92		1806.00 (262.50–3142.00)	
Score 9	12	27.3	289.22 (70.80–3829.00)		32.60 ± 3.55		1629.80 (81.84–1950.00)	
WHO grade								
Grade I^#^	4	9.1	9.1 (8.40–98.47)	0.003*	22.53 ± 2.65	< 0.001*	124.10 (62.00–1301.80)	0.194
Grade II	5	11.4	17.0 (9.40–4640.00)		24.68 ± 1.64		1738.33 (443.80–2154.89)	
Grade III	7	15.9	11.00 (8.00–44.30)		25.87 ± 3.30		2408.80 (371.40–2705.30)	
Grade IV	16	36.4	299.63 (8.60–975.00)		27.85 ± 2.92		1806.00 (262.50–3142.00)	
Grade V	12	27.3	289.22 (70.80–3829.00)		32.60 ± 3.55		1629.80 (81.84–1950.00)	
Metastasis								
Absent	17	37.8	10.00 (8.00–241.62)	< 0.001*	25.04 ± 4.08	< 0.001*	1513.70 (62.00–2705.30)	0.981
Present	28	62.2	328.13 (8.60–4640.00)		29.98 ± 3.51		1696.70 (81.84–3142.00)	

Min. minimum, *Max.* maximum, *SD* standard deviation, Mann Whitney test& Kruskal Wallis test.

P: Comparing the different categories, *: Significant when p value < 0.05, #: excluded from relation due to small size.

On the other hand, no significant association was found for FORα regarding Gleason scores, WHO grades and presence of metastasis among prostate cancer patients (*p* = 0.415, 0.194 and 0.981 respectively) as shown in Table 3.

### Correlations of serum total PSA, TK1 and FORα with different parameters among patients with prostate cancer

Using Spearman’s correlation coefficient, there were statistically significant positive correlations between total PSA with Gleason score and WHO grades (*P* < 0.001 and *P* = 0.001) respectively. Regarding TK1, our results exhibited significant positive correlations with Gleason score, WHO grades, and total PSA levels with p-value < 0.001 each. However, as shown in Table 4, no statistically significant correlations were found between FORα with any of the studied parameters.

**Table 4:** Correlations of serum total PSA, TK1 and FORα with different parameters among patients with prostate cancer.

	Total PSA		TK1		FORα	
	*R*	*P*	*R*	*P*	*R*	*P*
Age	0.013	0.930	− 0.051	0.738	− 0.026	0.863
Gleason score	0.509	< 0.001*	0.731	< 0.001*	− 0.025	0.873
WHO Grade	0.484	0.001*	0.747	< 0.001*	0.037	0.813
Total PSA	–	–	0.482	0.001*	0.030	0.844
TK1	–	–	–	–	0.041	0.792

*R* Spearman’s rho.

*P value < 0.05 is significant.

### Diagnostic accuracy of total PSA, TK1, and FORα

**Fig. 2:**
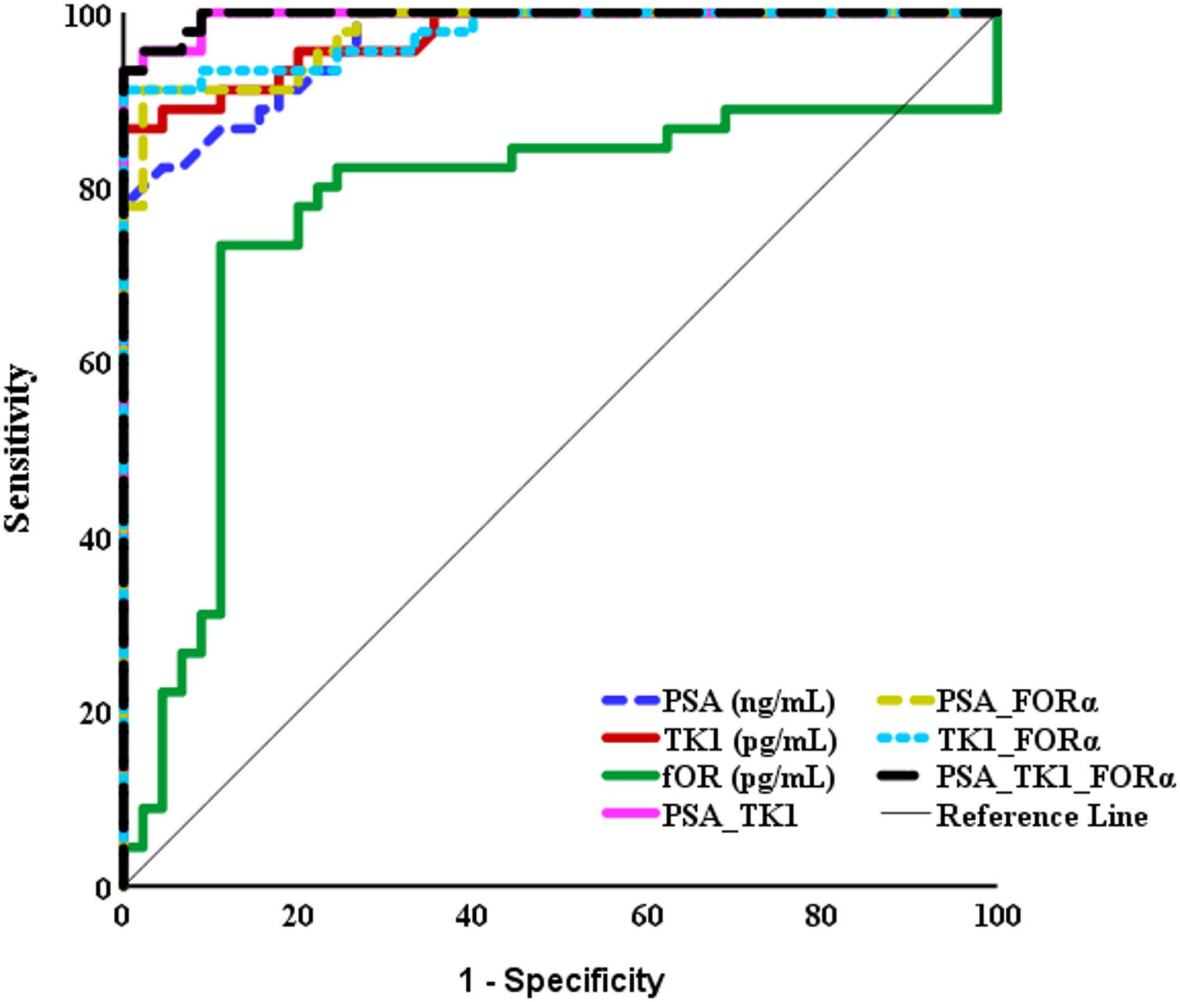
ROC Curve for total PSA, TK1, FORα and their combinations for discrimination between prostate cancer and the control group.

The diagnostic value of total PSA, TK1, and FORα were evaluated by ROC analysis. The results are shown in Figure 2. Total PSA and TK1 showed excellent discriminatory power, with high AUC values (0.966, 0.973 respectively), while FORα showed moderate discriminatory power with AUC = 0.770. The optimal cutoff values, sensitivities, specificities, and predictive values for each biomarker are provided in Table 5. Regarding combination of total PSA + TK1, the AUC was 0.996, which is higher than the AUC of either total PSA or TK1 alone, suggesting that this combination improves the discriminatory power, resulting in better differentiation between the two groups. In terms of combination of total PSA + FORα, the AUC was 0.976, which is higher than the AUC of FORα alone. Regarding combination of TK1 + FORα, the AUC was 0.976, similar to the AUC of the combination of total PSA + FORα. This indicates that both combinations (total PSA + FORα and TK1 + FORα) have comparable discriminatory power. As for combination of the three markers, results revealed an AUC of 0.996, which is similar to the combination of total PSA + TK1.

**Table 5:** Validity of total PSA, TK1 and FORα individually and in combination for discrimination between prostate cancer and the control group.

AUC	95% CI	*P*	Cut off	Sensitivity (%)	Specificity (%)	PPV (%)	NPV (%)	Accuracy (%)
Total PSA								
0.966	0.936–0.995	< 0.001*	> 9.6	82.22	95.56	94.88	84.31	88.89
TK1								
0.973	0.945–0.999	< 0.001*	> 22.1	91.11	88.89	89.13	90.91	90.0
FORα								
0.770	0.661–0.879	< 0.001*	> 918.16	73.33	88.89	86.84	76.92	81.11
Total PSA + TK1								
0.996	0.988–1.000	< 0.001*	–	95.56	97.78	97.73	95.65	96.67
Total PSA + FORα								
0.976	0.952–1.000	< 0.001*	–	88.89	97.78	97.56	89.80	93.33
TK1 + FORα								
0.976	0.950–1.000	< 0.001*	–	91.11	95.56	95.35	91.49	93.33
Total PSA + TK1 + FORα								
0.996	0.989–1.000	< 0.001*	–	95.56	97.78	97.73	95.65	96.67

*AUC* area under ROC curve, *CI* confidence interval, *PPV* positive predictive value, *NPV* negative predictive value.

*P value Significant < 0.05.

### Prediction of outcome

Logistic regression analysis was conducted for prediction of prostate cancer susceptibility, using total PSA, TK1, and FORα as covariates. Higher total PSA, TK1 and FORα were considered independent predictors for prostate cancer susceptibility either in univariate or multivariate analysis. For predicting the presence of metastasis among prostate cancer patients, higher PSA and TK1 levels were considered independent predictors for metastasis. Linear regression analysis for predicting Gleason score among prostate cancer patients was done. Among the variables examined, higher total PSA, TK1, and the presence of metastasis showed significant associations with Gleason score in the univariate analysis, while in the multivariate analysis, only higher TK1 and the presence of metastasis remain significant predictors for higher Gleason score. Data are summarized in Table 6.

**Table 6:** Logistic regression analysis for prediction of prostate cancer susceptibility.

	Univariate analysis for prediction of prostate cancer susceptibility			Multivariate analysis for prediction of prostate cancer susceptibility		
	*P*	OR	95% CI	*P*	OR	95% CI
Total PSA	0.009*	1.734	1.150–2.614	0.027*	1.021	1.010–1.036
TK1	0.001*	1.955	1.308–2.922	0.001*	1.114	1.059–1.172
FORα	< 0.001*	1.001	1.000–1.001	0.046*	1.009	1.003–1.031

	Univariate analysis for prediction of metastasis among patients			Multivariate analysis for prediction of metastasis among patients		
	*P*	OR	95% CI	*P*	OR	95% CI
Total PSA	0.002*	1.009	1.003–1.015	0.027*	1.007	1.001–1.012
TK1	< 0.001*	1.213	1.088–1.353	0.014*	1.108	1.067–1.269
FORα	0.657	1.000	1.000–1.001			

	Univariate analysis for prediction of Gleason score among patients			Multivariate analysis for prediction of Gleason score among patients		
	B		*P*	B		*P*
Total PSA	0.545		0.001*	0.176		0.267
TK1	0.154		< 0.001*	0.113		< 0.001*
FORα	0.224		0.485			
Metastasis	1.308		< 0.001*	0.997		0.001*

*OR* odd ratio, *CI* confidence interval, *B* unstandardized coefficients.

*Significant when p value < 0.05.

## Discussion

Prostate cancer is one of the most common malignancies in the male reproductive system with a mortality rate that is among the top five worldwide. Moreover, it is the second most frequently diagnosed cancer among men. Many patients could be curable if the disease is detected in the early stages. Unfortunately, most patients are diagnosed when the disease is metastatic, with a 5-year survival rate of approximately 30%, indicating the need for better diagnostic and predictive markers for prostate cancer^8^.

Against a backdrop of extensive research into various biomarkers for prostate cancer, the significance of developing non-invasive, sensitive, and specific diagnostic tools is increasingly recognized. TK1 and FORα, known for their elevated expression in cancerous states, were identified as potential candidates for this investigation^7,9^. TK1 is an enzyme involved in DNA synthesis and repair, and its levels are often elevated in rapidly dividing cells, such as cancer cells and has been studied as a potential biomarker for cancer diagnosis and monitoring treatment response^9^.

On the other hand, Folate receptor alpha (FORα) is a cell surface glycosyl-phosphatidylinositol (GPI) anchor glycoprotein encoded by the *FOLR1* gene found in chromosome 11. It binds to folate (vitamin B9) and is involved in the uptake of this essential nutrient. Folate receptor alpha is often overexpressed in cancer cells, as they have an increased need for folate to support their rapid growth and division^7^.

This study aimed to verify the applicability of these markers in improving the diagnostic accuracy for prostate cancer, reflecting a broader research agenda to enhance prognostic methodologies.

In this study, serum TK1 level was measured in prostate cancer patients and compared with the control group. TK1 level was significantly higher in the prostate cancer group with mean value 28.11 ± 4.41pg/ml versus 15.66 ± 5.89 pg/ml in control group (*P* < 0.001).

Previously, studies aligned with our findings that TK1 has the potential as a diagnostic biomarker for prostate cancer. Hanousková et al. (2020) reported that TK1 levels were elevated in all prostate cancer patients and even more so in those with severe forms of the disease. They found the diagnostic efficiency of TK1 to be 0.792, with a specificity of 53.6% and a sensitivity of 94.9%. Moreover, combining TK1 with PSA increased the diagnostic accuracy (AUC = 0.87)^10^. This finding underscores the importance of combining TK1 measurements with other diagnostic markers like PSA to enhance overall diagnostic accuracy.

The findings of the present study underscored the diagnostic potential of total PSA and TK1 as individual biomarkers for prostate cancer, demonstrating high Area Under the Curve (AUC) values of 0.966 and 0.973, respectively. Total PSA had a sensitivity of 82.22% and a specificity of 95.56%, while serum TK1 had a sensitivity of 91.11% and a specificity of 88.89%. These results confirm the established role of total PSA in prostate cancer screening and introduce TK1 as a similarly effective biomarker with excellent discriminatory power. The high AUC values indicate a robust ability to differentiate between prostate cancer patients and control groups, reflecting high sensitivity and specificity^11^.

However, a comprehensive meta-analysis found that the Area under the curve (AUC) for TK1 ranged from just above 0.7 to below 0.9, indicating moderate diagnostic efficacy. Consequently, the study concluded that TK1 alone is not a highly effective tumour marker. It suggests that combining multiple tumour markers may enhance diagnostic accuracy in identifying tumours^12^.

Our study demonstrated a significant association between serum TK1 levels and tumour aggressiveness in prostate cancer, as reflected by Gleason scores and WHO grade groups. Elevated TK1 levels with increasing Gleason scores suggested that TK1 may be responsive to cellular proliferation associated with more aggressive tumour pathology. The exceptionally high levels observed in Gleason score 9 could imply a pronounced proliferative activity, potentially valuable for distinguishing highly malignant tumours from less aggressive forms.

Li et al. (2018) aligned with our finding and observed that higher serum levels of TK1 are correlated with higher Gleason scores. This indicates that TK1 might be a useful biomarker for the aggressiveness of prostate cancer, as higher Gleason scores generally represent more advanced and aggressive forms of the disease^13^. In addition, another study found that TK1 expression levels were associated with tumour grade and Gleason score, suggesting that higher TK1 expression could be linked to more aggressive prostate cancers^14^.

Another research suggested that while exploring the Molecular Integrative Quantitative Gleason (MIQ-Gleason) system, there might be a correlation between TK1 and Gleason score, aiming to enhance the clinical utility of Gleason scoring^15^. Additionally, Řezáč et al. (2023) supported these findings, showing that TK1 levels differentiate prostate cancer patients from healthy controls and correlate with Gleason scores and other risk factors for advanced disease. They concluded that TK1 could be a valuable tumour marker for diagnosing and stratifying prostate cancer^16^.

In examining WHO grade groups, the clear trend of increasing TK1 levels with advancing tumour grade substantiates the enzyme’s capability to mirror the biological severity of prostate cancer. Also, it highlights TK1’s potential in discriminating between moderately aggressive and highly aggressive tumours, which is crucial for tailoring therapeutic approaches.

In addition, the statistical significance of higher TK1 levels in patients with metastasis versus those without suggests TK1’s role in signalling metastatic potential. This finding could lead to the application of TK1 as a prognostic marker for anticipating disease spread, thereby aiding in earlier intervention and management strategies^17^.

These findings were in accordance with Xie et al. (2022) study that had a general hypothesis stating that TK1 expression is significantly upregulated in prostate cancer and is associated with higher clinical and pathological stages. Notably, elevated mRNA expression of *TK1* is correlated with higher Gleason scores, higher clinical stages, and shorter survival, indicating its role in the progression of prostate cancer^18^.

Furthermore, our study identified TK1 as a significant independent predictor of prostate cancer susceptibility (OR = 1.213, 95% CI;1.088–1.353). This suggests that higher levels of TK1 are associated with an increased likelihood of developing prostate cancer. The role of total PSA and TK1 as prognostic markers is well-documented, indicating their utility in the clinical setting for identifying individuals at higher risk of prostate cancer^6,19^.

Lundgren et al. (2022) supported this finding and found that the estimated OR (adjusted for age) for dying from prostate cancer among the men who had a TK1 value in the upper tertile was 2.39 (95% confidence interval 1.02–5.63). Regardless of the cause of death, the corresponding OR was 2.81 (1.24–6.34). Hence, the authors concluded that high levels of TK1 predict death in prostate cancer within 30 years of follow-up^6^.

Further analysis concerning the presence of metastasis among prostate cancer patients indicated that higher levels of total PSA and TK1 serve as independent predictors. This finding is critical as it highlights these biomarkers’ potential in identifying patients at risk of developing metastatic disease, which is pivotal for tailoring treatment approaches and monitoring disease progression. In particular, the predictive value of TK1 aligns with its role in reflecting tumour dynamics and cellular proliferation, offering a valuable tool for assessing disease severity^11^.

This finding was consistent with a previous study by Murtola et al. (2020), which established that serum TK1 levels were significantly higher in men with de novo metastatic prostate cancer compared to those without metastasis, indicating a potential association between higher TK1 levels and more advanced cancer stage. The study also found that higher serum TK1 concentrations at diagnosis were associated with an increased risk of death from prostate cancer, suggesting that serum TK1 levels could serve as a prognostic marker for prostate cancer patients^4^.

Moreover, the exploring predictors of Gleason score identified total PSA, TK1, and the presence of metastasis as significant in the univariate model. However, in the multivariate analysis, only higher levels of TK1 and the presence of metastasis remained statistically significant. This distinction underscores the robustness of TK1 as a predictor of tumour aggressiveness, reflected by higher Gleason scores. Li et al. (2018) supported our finding and detected that serum TK1 concentration was a reliable prognostic biomarker for prostate cancer, associated with Gleason score, making it valuable in predicting disease severity and progression^13^.

The significance of metastasis presence further reinforces the correlation between advanced disease stages and higher tumour grades, suggesting these factors are crucial in understanding and managing patient outcomes^20^. Furthermore, recently, it was found that TK1 is identified as an independent variable of overall survival (OS) of prostate cancer, showing statistical significance when combined with total PSA levels. The predictive capacity of TK1 was highlighted by its ability to predict a difference of up to 10 years in OS, depending on patient subgroups, at a median of 9 years before prostate cancer diagnosis. The serum level of TK1 was found to be independent of all screening data, including PSA levels, indicating its unique predictive capacity. Minor variations in TK1 concentration significantly impacted OS, with the highest difference in survival probability of more than 10 years observed in 62-year-old men with varying TK1 concentrations. Univariate and multivariate logistic regression analyses revealed that TK1 and total PSA were significant predictors for OS, with TK1 showing a p-value of 0.018 and total PSA a p-value of 0.001^19^.

Additionally, Xie et al. (2022) found that TK1 serves as a prognostic predictor in prostate cancer, correlating with poor outcomes, higher tumour stages, and promoting cancer progression, suggesting its potential diagnostic and therapeutic significance^18^.

Regarding serum folate receptor alpha, it was significantly higher in the prostate cancer group than the control group with median values 1686.4 pg/ml and 437.2 pg/ml respectively (*P* < 0.001). Also, FORα exhibited moderate discriminatory power with an AUC of 0.770 (73.33% sensitivity and 88.89% specificity). This suggests that while FORα may be useful in certain contexts, it does not reach the high diagnostic accuracy of PSA or TK1 when used alone. Its moderate performance might limit its utility as a standalone biomarker; however, it could be considered in combination strategies.

Folate receptor alpha (FORα) has been used to detect and treat cancer for years. FORα has shown promise as a diagnostic marker for prostate cancer, particularly in cases where traditional markers like PSA are less conclusive. Lian et al. (2021) demonstrated that folate-receptor-positive circulating tumour cells (FR + CTCs) levels were significantly higher in prostate cancer patients than those with benign urinary diseases. Notably, FR + CTCs demonstrated higher diagnostic accuracy than traditional PSA testing in patients with low PSA levels (< 10 ng/ml) (0.871 vs. 0.857). The combined use of FR + CTCs and PSA further increased diagnostic efficiency (FR + CTCs, 0.912; total PSA, 0.857), suggesting that FORα could serve as a valuable early diagnostic marker in prostate cancer, especially for patients with uncertain PSA levels^21^. Hence, utilizing FR + CTCs as a diagnostic marker could potentially improve the accuracy and timeliness of prostate cancer diagnosis, enabling healthcare providers to initiate appropriate treatment sooner and improve patient outcomes.

Also, high expression of FORα in prostate cancer patients may suggest its use as an attractive therapeutical target. The high expression of FORα in malignant tumours makes it a potential target for anti-tumour drugs. Various strategies have been explored, including monoclonal antibodies, antibody-drug conjugate (ADC), FORα-specific CAR T, vaccines, small molecules, and folate-drug conjugate^22^.

However, the current study’s evaluation of serum FORα levels did not establish a significant correlation with the Gleason score among prostate cancer patients. This suggests that FORα may not be sensitive to the histopathological variations within prostate tumours as indicated by the Gleason scoring system. The lack of association might imply that FORα is not influenced by the architectural features that the Gleason score assesses, which primarily reflect tumour differentiation rather than metabolic or proliferative activity directly.

The investigation into the relationship between FORα levels and WHO grade groups revealed variability across the spectrum, yet these differences did not reach statistical significance. The wide range of FORα levels observed across different grades, such as the notably high levels in Grade III compared to others, might reflect underlying biological variability or heterogeneity within tumour expressions of FORα. However, the absence of statistically significant differences suggests that FORα might not reliably differentiate between the aggressiveness of prostate cancer as categorized by WHO grades.

Furthermore, the analysis of FORα levels in metastatic presence showed no significant difference between patients with and without metastasis. This outcome indicates that while FORα might be upregulated in certain cancer types, its role in indicating metastatic potential in prostate cancer is limited^23,24^. This aligns with studies suggesting that the expression of FORα might be more pronounced in specific tumour types like ovarian and cervical cancers but less so in prostate cancer^24^.

These results suggest that FORα, despite its potential as a biomarker in other cancers, may not serve as a reliable indicator of tumour grade, Gleason score differentiation, or metastatic status in prostate cancer. The implications of these findings contribute to the broader understanding of biomarker specificity and the complexity of their application in oncological diagnostics.

Significantly, the combination of biomarkers has enhanced diagnostic accuracy beyond individual markers in our study. The combination of PSA + TK1 resulted in an AUC of 0.996 (95.56% sensitivity and 97.78% specificity), indicating almost perfect discrimination. This suggests a synergistic effect when these markers are used together, potentially allowing for a more refined screening strategy that could reduce false positives and negatives in prostate cancer diagnosis^6^.

Similarly, the combination of total PSA + FORα and TK1 + FORα achieved an AUC of 0.976, improving upon the AUC for FORα alone. This enhancement in discriminatory power highlights the benefit of biomarker panels in capturing a broader biological spectrum of tumour activity, thereby improving diagnostic accuracy.

The highest AUC observed was 0.996, from the combination of total PSA + TK1 and the trio combination of total PSA + TK1 + FORα. This demonstrates that combining total PSA + TK1 could offer the best strategy for accurately distinguishing between prostate cancer and non-cancerous conditions. This approach maximizes the strengths of each biomarker, covering different aspects of tumour biology, which could greatly improve early detection and potentially aid in personalized treatment planning^12^.

In conclusion, this study has significantly contributed to the understanding of the roles played by total PSA, TK1, and FORα as biomarkers in the diagnosis and prognosis of prostate cancer. The findings demonstrated that PSA and TK1, individually and in combination, possess strong diagnostic capabilities, effectively distinguishing between prostate cancer patients and control groups. Furthermore, their predictive power extends to assessing the likelihood of metastasis and the severity of the disease as indicated by the Gleason score. Although FORα showed moderate discriminatory power individually, its utility alongside other biomarkers underscore the potential of a multifaceted biomarker approach in enhancing diagnostic accuracy and prognostic assessments. These insights not only support the integration of these biomarkers into clinical practice but also suggest directions for future research aimed at optimizing treatment strategies and improving patient outcomes. By advancing our understanding of biomarker profiles in prostate cancer, this research contributes to the broader goal of tailored and precise medical interventions by targeting thymidine kinase which may offer a novel approach for treatment in this type of patients, ensuring better care and prognosis for patients. Also, FORα may have a beneficial role in drug delivery systems for FORα positive patients. However, our study has some limitations that should be avoided in future research. Upcoming studies should be multicentric with a larger sample size, using molecular techniques for better verification of results. All pathological subtypes of prostate cancer should be included as well as benign prostatic hyperplasia as a separate group. The studied markers should be compared with the patient’s follow up to assess disease free and overall survival.

## Data Availability

The datasets used and/or analyzed during the current study are available from the corresponding author on reasonable request.
